# Association of thrombosis and mortality in patients with COVID-19 infections: a hospital-based observational study

**DOI:** 10.1186/s43162-022-00153-5

**Published:** 2022-08-19

**Authors:** Sher M. Sethi, Sadaf Hanif, Madiha Iqbal

**Affiliations:** Internal Medicine, Depatment of Medicine, Aga Khan University Hospital Karachi, Stadium Road, Gulshan-e-Iqbal, Karachi, 74800 Pakistan

**Keywords:** COVID-19, COVID-19-associated coagulopathy, Pulmonary embolism, Thrombosis, Thromboembolism

## Abstract

**Objective:**

A hospital-based cross-sectional study on COVID-19 confirmed patients was conducted at the Aga Khan University Hospital, Karachi, Pakistan, from April to June 2021. Presence of thrombosis in these patients was compared with mortality. Platelet counts and D-dimer was also compared among survivor and non-survivor to identify the marker for severity of the disease.

**Results:**

Sixty-six patients were enrolled in the study and the mean age of the patients was 62.3 years and 45 patients (68.2%) were male. Pulmonary embolism was identified in 32 patients (48.5%) while non-pulmonary thrombosis occurred in 5 of the admitted patients (7.6%). In our study, mortality occurred in 34 patients (51.5%). Pulmonary embolism was identified in 20 recovered patients (62.5%) and 10 patients died (*p* value 0.03). Four patients (80%) with non-pulmonary thrombosis were non-survivors (*p* value 0.05). Median platelets were 73 in non-survivors and 109.5 in survivors (*p* value < 0.01). Both the groups had a median D-dimer of 3.8 (*p* value 0.024).

**Conclusion:**

Based on our study, we conclude that COVID-19 infection has the potential to cause hypercoagulable states. It increases the risk of thrombosis and with thrombosis it has a higher mortality rate. Thrombocytopenia is a biomarker with an adverse prognosis in these patients.

## Introduction

In 2019, the first case of acute respiratory distress syndrome due to SARS CoV-2 virus was identified in China. Due to the rapid global spread of the virus and increasing mortalities, it was declared as a pandemic by WHO in March 2020 [1]. Respiratory failure due to COVID-19 infection is most common manifestation of the illness and probably the most devastating one as well, but over time extra-pulmonary complications have also been identified [2]. Some of such extra-pulmonary thrombotic complications include stroke or myocardial ischemia, acute kidney injury, hepatocellular injury, and hematological complications [3].

Thrombotic complications and coagulopathies had greatly added to increased morbidity and mortality and prolonged hospitalization [4]. Coagulation abnormalities have been reported in COVID-19 patients and have been associated with significant mortality and morbidity. In fact now there is a separate entity that is being used for coagulopathic complications in such patients that is called COVID-19-associated coagulopathy [5]. Various studies have used coagulation parameters to identify the increased risk of death in COVID-19 patients [6].

The rationale for conducting this study was to identify the association of thrombosis and mortality in our population. This association can help us to consider thrombotic complications in our critically ill COVID-19 patients. The secondary aim was to identify D-dimer and platelet count as a marker of severity of the disease.

## Methods

This is a hospital-based retrospective observational study conducted at the Aga Khan University Hospital, Karachi, Pakistan, from April to June 2021 to identify the association of thrombosis and mortality in patients with COVID-19 infection. Aga Khan University Hospital is a pioneer tertiary care hospital with 650 beds and has Joint Commission International Accreditation (JCIA).

All patients who were aged > 18 years admitted to the hospital with positive reverse transcriptase-polymerase chain reaction for SARS-CoV-2 were included. Patients with any coagulation disorder, auto-immune disorder, pregnancy, and malignancy were excluded from this study. Each patient’s medical records were retrospectively reviewed if they met the inclusion criteria.

Demographics that include age and gender were recorded. There were two subcategories of age: patients aged 55 or less, and those aged 56 or older. Severity of COVID-19 infection was classified according to diagnosis and treatment protocol for Novel Coronavirus Pneumonia (Trial Version 7) [7]. It describes patients with critical illness who develop hypoxic respiratory failure, shock, and/or multiple organ failure. Patients with severe disease are those who require additional oxygen support or show greater than 50% lung involvement on radiological imaging. Mild disease are those with mild clinical symptoms without radiological evidence of pneumonia and moderate disease are those who have clinical symptoms with radiological evidence.

Use of mechanical ventilation, presence of pulmonary and non-pulmonary thrombosis was noted. Presence of thrombosis was confirmed via radiological imaging. Laboratory parameters that include coagulation profile and platelet counts were noted. Prothrombin and activated partial thromboplastin time was recorded at the time of admission. D-dimer and fibrinogen levels were monitored and their maximum level during their in-patient stay was recorded. Platelet counts were also evaluated and the lowest platelet count during their hospital stay was noted. Primary outcome was to identify association between thrombosis and mortality. Secondary outcome was to compare coagulation profile and platelets between survivors versus non-survivors.

Institute’s ethical review committee reviewed the study protocol and ethical exemption was granted (ERC Number: 2021–6419-18,237). Clinical data was filled by the investigators using a designed pro-forma and was then entered into the system software. Data analysis was done through IBM Statistical Package for the Social Sciences (SPSS) Version 26. Mean and standard deviation were computed for quantitative variables and frequency and percentage for categorical variables. Frequency of categorical variables were compared using the chi square test with a level of significance of 95%. Mann–Whitney *U* test was used to compare median and interquartile range between survivors and non-survivors with a level of significance of 0.05.

## Results

A total of 94 patients with a positive reverse transcriptase polymerase chain reaction for COVID-19 were admitted to our tertiary care hospital. Sixty-six patients were enrolled in the study and 28 patients were excluded from the study as per exclusion criteria. The mean age of the patients was 62.3 years and 45 patients (68.2%) were male. Most of our study population was 56 years and over the age of 56 (48 patients; 72.7%). Fourteen patients (21.2%) had moderate disease while 44 patients (66.7%) had severe COVID-19 infection. Mechanical ventilation was applied to 12 patients (18.1%). Pulmonary embolism was identified in 32 patients (48.5%) while non-pulmonary thrombosis occurred in 5 of the admitted patients (7.6%). In our study, mortality occurred in 34 patients (51.5%). Table 1 shows the demographic and clinical characteristics of the patients admitted with COVID-19 infection.

**Table 1:** Demographics and clinical characteristics of COVID-19 patients (*n* = 66)

	**Frequency (*n*)**	**Percentage (%)**
**Mean age**	62.3 years (SD + 13.3 years)	
**Age**		
55 years or less	18	27.3
56 years and above	48	72.7
**Sex**		
Male	45	68.2
Female	21	31.8
**Disease severity**		
Mild to moderate	8	12.1
Severe	14	21.2
Critical	44	66.7
**Mechanical ventilation**		
Yes	12	18.1
No	54	81.8
**Pulmonary embolism**		
Yes	32	48.5
No	34	51.5
**Non-pulmonary thrombosis**^a^		
Yes	05	7.6
No	61	92.4
**Clinical outcome**		
Mortality	34	51.5
Recovered	29	43.9
LAMA^b^	03	4.5

^a^Non-pulmonary thrombosis: ischemic stroke (2 patients), arterial thrombosis (2 patient), Acute Coronary syndrome/LV thrombus (1 patient)

^b^Leaving against medical advice

While comparing the clinical outcome, 10 patients (55.6%) with an age group of 55 years and less were recovered from their illness while 27 patients (56.3%) with age above 55 years had mortality (*p* value 0.39). In our study population, mortality occurred more in male gender (*n* = 25, 55.6%) when compared with female gender (*p* value 0.25). Most of our patients (*n* = 27, 61.4%) had a severe COVID-19 infection and were expired. Whereas 62.5% of patients with mild disease and 64.3% of patients with moderate disease recovered from their COVID-19 infection (*p* value: 0.09). Ten patients (83.3%) requiring mechanical ventilation had mortality (*p* value 0.03). Pulmonary embolism was identified in 20 recovered patients (62.5%) and 10 patients died (*p* value 0.03). Four patients (80%) with non-pulmonary thrombosis were non-survivors (*p* value 0.05). Table 2 demonstrates comparison of disease outcomes on the basis of basic demographic characteristics, coagulation profile, and disease severity.

**Table 2:** Comparison of disease outcomes among PCR confirmed COVID-19 patients on the basis of basic demographic characteristics, coagulation profile, and disease severity (*n* = 66)

	**Clinical outcome**			*p* value^**^
	**Mortality (*n* = 34)**	**Recovery (*n* = 29)**	LAMA* (*n* = 3)	
	**Frequency (%)**			
**Age**				
55 years or less	07 (38.9)	10 (55.6)	01 (5.5)	0.39
56 years and above	27 (56.3)	19 (39.5)	02 (4.2)	
**Sex**				
Male	25 (55.6)	17 (37.7)	03 (6.7)	0.25
Female	09 (42.9)	12 (57.1)	0	
**Disease severity**				
Mild to moderate	02 (25)	05 (62.5)	01 (12.5)	0.09
Severe	05 (35.7)	09 (64.3)	0	
Critical	27 (61.4)	15 (34.1)	02 (4.5)	
**Mechanical ventilation**	10 (83.3)	02 (16.7)	0	0.03
**Pulmonary embolism**	10 (31.3)	20 (62.5)	02 (6.2)	0.03
**Non-pulmonary thrombosis**	04 (80.0)	0	01 (20.0)	0.05
**Steroids**	34 (52.3)	28 (43.1)	03 (4.6)	0.48
**Tocilizumab**	07 (46.7)	07 (46.7)	01 (6.6)	0.95
**Remdesivir**	24 (57.1)	16 (38.1)	02 (4.8)	0.44

^*^Leaving against medical advice

^**^Chi-square test of significance was applied

Comparison of coagulation profile showed a median prothrombin time of 14.6 (interquartile range 4) in non-survivors while 13.7 (interquartile range 11) in the survivor group (*p* value 0.01). Similarly, median activated partial thromboplastin time in non-survivors was 30.7 (interquartile range 12) and in survivors was 22.3 (interquartile range 11) (*p* value 0.00). Median platelets were 73 in non-survivors and 109.5 in survivors (*p* value < 0.01). Both the groups had a median D-dimer of 3.8 (*p* value 0.024). Table 3 shows the detailed comparison of serum platelets and coagulation profile with disease outcome.

**Table 3:** Comparison of disease outcomes among COVID-19 patients on the basis of serum platelets and coagulation profile (*n* = 63)

	**Median (IQR)**		***p* value**^a^
	**Non-survivor (*n* = 34)**	**Survivor (*n* = 29)**	
**PT**	14.6 (4)	13.7 (11)	0.01
**INR**	1.4 (0)	1.35 (1)	0.01
**APTT**	30.7 (12)	22.3 (11)	0.00
**Platelets**^b^	73.0 (61)	109.5 (159)	< 0.01
**Fibrinogen**	188 (544)	213.5 (5)	1.00
**D-dimer**	3.8 (8)	3.8 (5)	0.24

*IQR* Interquartile range, *PT* Pro-thrombin time, *INR* International normalized ratio, *APTT* Activated partial thromboplastin time

^a^Mann-Whitney *U* test

*p* value of 0.05 or less is significant

^b^8 patients have chronic liver disease

## Discussion

Our observational study reveals that patients with COVID-19 infection who develop thrombosis have higher mortality. Majority of our study participants were elders (age 56 and above) and had male predominance. Critical COVID-19 infection was more prevalent in our hospitalized patients. Almost half of the patients developed pulmonary embolism. Most of the patients with pulmonary embolism survived while patients with non-pulmonary thrombosis had mortality. Thrombocytopenia was the major predictive marker associated with mortality.

COVID-19 infection had gained more importance because of its variable clinical features. It was initially considered as a disorder of the respiratory system causing pneumonia and acute respiratory distress syndrome [8]. Despite this, it had been associated with an increased risk of arteriovenous thrombosis. A variety of non-pulmonary thrombotic complications were detected in the spleen, legs, heart, and brain [9]. The most common thrombosis identified with COVID-19 infection is pulmonary embolism and deep venous thrombosis [10]. Non-pulmonary thrombosis has also gained momentum and has been reported with COVID-19 infection and includes acute limb [11], cerebrovascular accident [12], and acute coronary syndrome [13]. Our study also identified that 37 patients (56%) had thrombotic events during their COVID-19 infection illness.

The prevalence of thrombosis with COVID-19 infection is not well-known [10]. Klok et al. reports pulmonary embolism as a major thrombotic event with an incidence rate of 87% [14]. While Bompard et al. reports 50% incidence rate of pulmonary embolism [15]. We also report in our study that 32 (48.5%) of our patients had pulmonary embolism.

Pulmonary embolism with COVID-19 infection is termed as a poor prognostic indicator. A systemic review and meta-analysis had reported a 45.1% mortality rate in COVID-19 patients with pulmonary embolism [16]. Despite the fact that another meta-analysis concludes that though there is more prevalence of pulmonary embolism with COVID-19, it does increase risk of mortality in critically ill COVID-19 patients [17]. Interestingly, the overall mortality rate in our study participant with pulmonary embolism was 31.3% while 62.5% patients with pulmonary embolism recovered (*P* value 0.03).

As COVID-19 surge continues, various non-pulmonary thrombotic events were identified [18]. It accounts for 7.2% of COVID-19 patients with acute coronary syndrome [19]. Mao et al. report 3% of their COVID-19 patients developing an ischemic stroke [20]. An incidence rate of arterial thrombosis with COVID-19 infection is around 4.4% [21]. In our study, we identified five different cases of non-pulmonary thrombosis (7.5%). Cerebrovascular accident was identified in two individuals in which one patient had left middle cerebral artery infarct while the other patient had dural venous sinus thrombosis. Two of our patients presented with acute limb ischemia while one patient had non-ST elevated myocardial infarction with evidence of left ventricular thrombus on echocardiography.

The mortality rate of COVID-19 patients with arterial thrombosis is estimated at 20% [21]. An in-hospital mortality of 34.4% is reported in COVID-19 patients with ischemic stroke [22]. The International Prospective Registry of Acute Coronary Syndromes in Patients with COVID-19 shows a higher mortality of around 23% [23]. We had a higher mortality (80%) than reported in the past. We hypothesize that it could be due to delayed presentation, critical illness, multi-organ involvement, and extensive thrombosis along with COVID-19 infection.

Thrombocytopenia with COVID-19 infection is common. It is postulated that thrombocytopenia in patients with COVID-19 infection is caused by reduced platelet production, increased platelet destruction and increases platelet consumption [24]. Thrombocytopenia is a marker with an unfavorable prognosis. COVID-19 patients with platelet count of 100 – 150 × 10^9^/μL had a relative risk of 3.4 for in-hospital mortality which increased to 9.9 and 13.6 if platelet count are 50 − 100 × 10^9^/μL and < 50 × 10^9^/μL [25]. Our study also identified thrombocytopenia in our study participants with median count of 73 × 10^9^/μL in non-survivor vs. 109 × 10^9^/μL in survivor (*P* value < 0.001). 8 of our patients have chronic liver disease which was the possible pre-existing factor for thrombocytopenia. All patients had their malaria antigen and dengue antigen or serology tested at the hospital to rule out other infections that cause thrombocytopenia.

Level of D-dimer is always taken into consideration when thrombosis is diagnosed. It is said that a 3 to fourfold increase in D-dimer levels is associated with a poorer outcome [26]. Yao et al. reports in their study on COVID-19 patients that elevated D-dimer correlates with severity of the disease and is a marker for mortality [1]. On the other hand, a recent study reports that D-dimer levels have a limited role in prognostication of the disease [27]. It is surprising that in our study, when evaluating D-dimer levels, we found the same median level of 3.8 in both survivors and non-survivors. However, this result is not statistically significant (*P* value 0.24).

There are certain limitations in our study. Firstly, it is a single-centered retrospective study and cannot be generalized to the whole population. Secondly, the sample size is small and requires more future studies to validate this association. Thirdly, management of thrombosis was not discussed because majority of the patients who were started on anticoagulation were stopped in between their hospital stay due to thrombocytopenias.

The strength of our study is that it compared not only pulmonary but also non-pulmonary thrombosis with mortality. Non-pulmonary thrombosis has a more adverse outcome than pulmonary thrombosis. Beside this, we also identified that thrombocytopenia (platelet counts less than 100 × 109/μL) was associated with poor outcome. D-dimer was not a significant marker in evaluating the severity of illness in our study. Future meta-analyses and trials are necessary to strengthen these evidences and to evaluate the management strategies for these patients.

## Conclusion

Based on our study, we conclude that COVID-19 infection has the potential to cause hypercoagulable states. It increases the risk of thrombosis and with thrombosis, it has a higher mortality rate. Thrombocytopenia is a biomarker with an adverse prognosis in these patients.

## Data Availability

Data sharing is not applicable to this article as no datasets were generated or analyzed during the current study.
